# How do young chinese physicians cope with stress under standardized training? Investigating perceived stress, depression, and their interrelationships

**DOI:** 10.1371/journal.pone.0356791

**Published:** 2026-08-24

**Authors:** Yanhong Jiang, Bao Tian, Lei Yan, Hui Chen, Wenzheng Peng, Bo Wu, Qian Su, Pingting Yang

**Affiliations:** 1 Health Management Medical Center, The Third Xiangya Hospital, Central South University, Changsha, Hunan, China; 2 Health Management Center, First Affiliated Hospital of Jishou University, JiShou, Hunan, China; 3 Post Graduation Medical Education Office, The Third Xiangya Hospital, Central South University, Changsha, Hunan, China; 4 Department of General Practice, The Third Xiangya Hospital, Central South University, Changsha, Hunan, China; 5 Department of Otolaryngology-Head and Neck Surgery, The Third Xiangya Hospital, Central South University, Changsha, Hunan, China; 6 Department of Orthopedics, The Third Xiangya Hospital, Central South University, Changsha, Hunan, China; University of Trieste: Universita degli Studi di Trieste, ITALY

## Abstract

Resident physicians undergoing standardized residency training (SRT) in China face substantial occupational stress, yet the extent of their psychological burden and the pathways linking stress to depressive symptoms remain incompletely characterized. This multicenter, cross-sectional study examined the psychological burden and its underlying pathways among 504 resident physicians in China’s SRT system. Participants from teaching hospitals in southern China completed validated questionnaires assessing perceived stress (PSS-10), burnout (MBI-GS), effort–reward imbalance (ERI), and depressive symptoms (PHQ-9), and the data were analyzed using path analysis with bias-corrected bootstrapping to test for serial mediation. The prevalence of psychological problems was high: 38.49% reported high perceived stress, 83.13% experienced burnout (37.5% moderate or severe), 30.16% exhibited effort–reward imbalance, and 27.58% screened positive for probable depression. Mean scores were 17.61 ± 6.36 for perceived stress, 43.26 ± 13.04 for burnout, 0.94 ± 0.65 for ERI, and 7.83 ± 6.47 for depressive symptoms. All four variables were significantly and positively correlated (ρ = 0.338–0.631). The path analysis indicated a serial mediation pattern: higher perceived stress was associated with higher ERI (β = 0.43), higher ERI with greater burnout (β = 0.49), and greater burnout with more severe depressive symptoms (β = 0.37); perceived stress was also directly associated with depressive symptoms (β = 0.21). In conclusion, resident physicians in China experience a substantial psychological burden, characterized by a sequential association linking stress, effort–reward imbalance, burnout, and depressive symptoms. Because the design was cross-sectional, these pathways reflect associations rather than causal effects. Safeguarding trainee well-being and healthcare quality will require targeted interventions that reduce effort–reward imbalance through improved compensation and job security, mitigate burnout through workload management, and ensure access to mental health resources.

## 1. Introduction

Physician mental health is a global public health priority. Launched nationwide in 2014, China’s SRT follows a “5+3” model: five years of undergraduate medical education followed by three years of residency that is now mandatory for independent clinical practice. Trainees rotate through multiple departments and must pass both continuous in-training assessments and a national graduation examination (comprising theoretical and OSCE-based skills components). Residents enter through three pathways that differ markedly in job security: clinical professional master’s students (who complete residency and a research degree concurrently), commissioned residents (employed by a sponsoring institution to which they return), and “socialized” residents (who have no guaranteed employer and face uncertain post-training employment). Workloads are heavy, commonly exceeding 40–60 hours per week with frequent night shifts, and the predominant stressors differ by training year—first-year residents face role adaptation, whereas third-year residents confront graduation examinations and job-market pressures [1]. Residents undergoing SRT face severe psychological challenges: studies indicate that approximately one-third experience depression, anxiety, or burnout [2]. These challenges are compounded by systemic stressors within the Chinese healthcare context, including heavy clinical workloads, long working hours, academic pressure, financial strain, and job insecurity [3–5]. The cumulative burden of these stressors places residents at high risk of psychological distress, which can compromise both personal well-being and the quality of patient care [6,7].

Perceived stress—the subjective sense of imbalance between demands and coping capacity [8]—is highly prevalent among healthcare workers and is a key risk factor for adverse mental and physical health outcomes [9,10]. For resident physicians, who are at a critical developmental stage, perceived stress is particularly salient and may be an important correlate of psychological morbidity.

Perceived stress does not operate in isolation but is closely linked to other occupational and psychological constructs. It is strongly associated with occupational burnout, a state of emotional exhaustion and reduced accomplishment that arises from chronic workplace stress [11,12], and burnout is itself a recognized risk factor for medical errors and physician attrition [13]. In parallel, the effort–reward imbalance (ERI) model offers a useful framework for understanding how occupational stress arises from a mismatch between high work effort and low reward, a condition common in medical training [14,15]. Both burnout and ERI are recognized as important mediators and outcomes of workplace stress.

A key outcome associated with these stressors is depression. Resident physicians are at substantially greater risk of depression than the general population [16], with pooled prevalence estimates as high as 28.8% [17]. In this group, depression affects not only individual health but also the quality of patient care [18].

Although the high prevalence of perceived stress, burnout, ERI, and depression among residents is well documented [2,12,17,19,20], important gaps remain. Few studies have specifically examined how these factors are interconnected within the particular context of China’s SRT system, and the pathways through which perceived stress may relate to depression—potentially by way of burnout and ERI—are not fully understood. Clarifying these interrelationships is essential for developing effective, targeted interventions that move beyond describing the problem to addressing its underlying mechanisms.

This study therefore investigated the interrelationships among perceived stress, burnout, ERI, and depressive symptoms in Chinese resident physicians. We hypothesized that (1) these four variables would be significantly associated with one another, and (2) burnout and ERI would sequentially mediate the relationship between perceived stress and depressive symptoms. By testing this serial mediation model, we sought to provide evidence for a mechanistic pathway that could inform more precise strategies to support resident well-being.

## 2. Methods

### 2.1. Study design

A cross-sectional survey was conducted to assess perceived stress, occupational burnout, ERI, and depressive symptoms among resident physicians at four hospitals in southern China. The study followed the Strengthening the Reporting of Observational Studies in Epidemiology (STROBE) reporting guidelines [21]. Ethical approval was granted by the Ethics Committee of the Third Xiangya Hospital of Central South University (No. 2024-S386). Electronic informed consent was obtained from all participants: before accessing the survey, participants were shown an informed-consent page describing the study and could proceed only after indicating their agreement.

### 2.2. Setting

An online survey was conducted via the Wenjuanxing (Sojump) platform from April 1 to April 20, 2025. Invitations were sent to approximately 1,756 eligible residents, of whom 561 submitted the survey, corresponding to a participation rate of about 32% (561/1,756). To reduce careless responding, submissions completed in under 200 seconds were excluded; for this 69-item questionnaire, that threshold corresponds to fewer than 2.9 seconds per item—below the minimum time needed to read and answer each item attentively—and response times below approximately two seconds per item are widely used to flag invalid “speeded” responses [22,23]. Applying this a priori rule excluded 57 submissions, yielding 504 valid questionnaires and a valid-response rate of 89.84% (504/561).

### 2.3. Participants and enrollment

**Fig 1:**
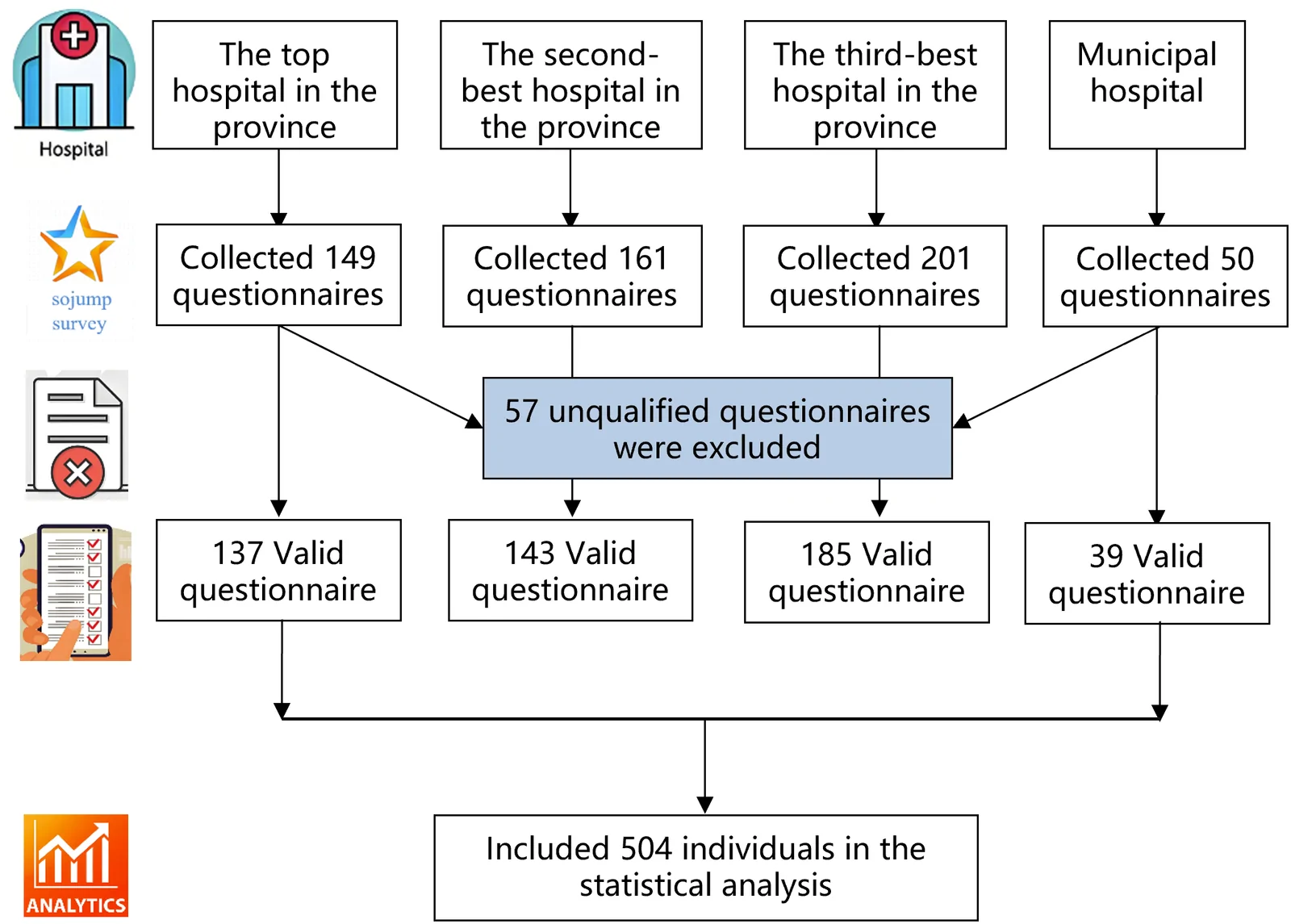
Questionnaire Collection Flowchart.

Four hospitals—three major teaching hospitals and one municipal hospital—served as recruitment sites. The target number of participants was allocated proportionally across sites according to the number of resident physicians at each institution, as outlined in Fig 1. Only residents currently enrolled in standardized training programs were eligible; those not enrolled were excluded. Eligible residents were invited to complete the questionnaire through WeChat groups or by in-person contact with members of the research team.

### 2.4. Questionnaire and data collection

A questionnaire comprising five sections was administered to assess the variables of interest, as described below.

#### 2.4.1. Part One (Demographic characteristics).

This section collected participants’ demographic characteristics, including sex, age, training specialty, residency category, affiliated hospital, current training year, highest academic degree, marital status, undergraduate institution type, and whether they had passed the national medical licensing examination.

#### 2.4.2. Part Two (Depressive symptoms).

Depressive symptoms were assessed with the Patient Health Questionnaire-9 (PHQ-9), which comprises nine items, each rated on a 4-point Likert scale (0 = not at all, 1 = several days, 2 = more than half the days, 3 = nearly every day), giving total scores from 0 to 27. A score ≥10 indicates clinically significant (moderate or greater) depressive symptoms based on the established screening threshold. Because the PHQ-9 is a screening instrument, a positive screen indicates probable depression and does not constitute a clinical diagnosis of major depressive disorder. The PHQ-9 was selected for its brevity, its self-administration format (suitable for online delivery), and its status as the most extensively validated depression screener internationally and in China, with established reliability and criterion validity among Chinese healthcare workers and physicians [24–26]. Previous validation studies have reported robust diagnostic performance, with 88% sensitivity and 88% specificity for detecting depressive symptoms [24,25]. Cronbach’s α has been reported as 0.92 for physicians and 0.88 for the general population [26]; in the current sample, it was 0.94.

#### 2.4.3. Part Three (Perceived stress).

Perceived stress was assessed with the 10-item Perceived Stress Scale (PSS-10), originally developed by Cohen [27], to measure individuals’ subjective appraisal of unpredictable and overwhelming demands in daily life. The scale contains six negatively phrased items and four positively phrased items that are reverse-scored. Participants responded on a 5-point Likert scale ranging from “never” (0) to “very often” (4), giving total scores from 0 to 40. Established cutoffs define three stress levels—low (0–13), moderate (14–19), and high (≥20)—with higher scores indicating greater perceived stress. We used the validated Chinese version of the PSS-10, which has demonstrated good reliability and validity (Cronbach’s α = 0.81) [28,29]; in the current sample, Cronbach’s α was 0.83.

#### 2.4.4. Part Four (Occupational burnout investigation).

Work-related burnout was assessed with the Maslach Burnout Inventory–General Survey (MBI-GS), originally developed by Maslach in 1996 [30] and adapted for use in China by Li Chaoping and Shi Kan [31,32]. The MBI-GS is widely used to measure occupational burnout and contains three subscales: emotional exhaustion (Ex, 5 items), cynicism (Cy, 5 items), and reduced personal accomplishment (PA, 6 items). Items are rated on a 7-point frequency scale ranging from 0 (never) to 6 (daily). Previously reported Cronbach’s α values were 0.88 (Ex), 0.83 (Cy), and 0.82 (PA); in the current sample, the subscale values were 0.92 (Ex), 0.87 (Cy), and 0.91 (PA). Burnout was scored using the cutoffs of Ye Zhihong [33] (exhaustion ≥25, cynicism ≥11, and reduced personal accomplishment ≥16), and cases were classified according to the framework of Li Yongxin [34]: none (no dimension above threshold), mild (one dimension), moderate (two dimensions), and high (all three dimensions above threshold).

#### 2.4.5. Part Five (Effort-Reward Imbalance).

Occupational stress arising from the effort–reward relationship was assessed with the Effort–Reward Imbalance (ERI) scale, developed by Siegrist in 1996 and introduced into China by Li Jian in 2004 [14,15]. The scale comprises 23 items across three components: effort (6 items), reward (11 items), and over-commitment (6 items). The effort score ranges from 6 to 30, the reward score from 11 to 55, and the over-commitment score from 6 to 24. The ERI ratio is calculated as E/(R × c), where E is the total effort score, R is the total reward score, and c is a correction factor equal to the ratio of the number of effort items to the number of reward items. An ERI > 1 indicates a high-effort–low-reward (imbalance) state, whereas an ERI ≤ 1 indicates low effort relative to reward. Previously reported Cronbach’s α values for the three components were 0.89, 0.89, and 0.64, indicating acceptable reliability [35]. In the current sample, the corresponding values were 0.92, 0.93, and 0.83.

### 2.5. Sample size

The adequacy of the final sample (N = 504) was evaluated with respect to the specified serial-mediation model rather than a simple item-count rule. The model estimated 10 free parameters (six structural paths, one exogenous variance, and three residual variances), yielding a participant-to-parameter ratio of approximately 50:1—far exceeding the 20:1 ratio recommended for path and structural equation models [36] and the minimum sample sizes indicated by Westland [37] and Wolf et al. [38] for models of comparable complexity. A bootstrap power analysis (2,000 resamples) indicated power >0.99 to detect the hypothesized serial indirect pathway (perceived stress → ERI → burnout → depressive symptoms) at N = 504, consistent with all structural paths and all bootstrap indirect effects reaching statistical significance (Table 6).

### 2.6. Statistical analysis

Analyses were performed using IBM SPSS 24.0 and Amos 26.0. Continuous variables were summarized as mean ± standard deviation (SD) and categorical variables as frequencies and percentages. The normality of the four scale scores was assessed using the Shapiro–Wilk test; all were non-normally distributed. Accordingly, the Mann–Whitney U test and the Kruskal–Wallis H test were used to compare scores across demographic groups, and Spearman rank-order correlations were used to examine the bivariate associations among perceived stress, burnout, ERI, and depressive symptoms. Multiple linear regression models adjusted for sociodemographic variables were then used to further examine these associations. Finally, a path model—a just-identified structural model among the four composite scores—was estimated to examine the direct and indirect associations among perceived stress, occupational burnout, ERI, and depressive symptoms. Indirect (mediation) effects were tested using bias-corrected bootstrap resampling (2,000 resamples), with 95% confidence intervals (CIs) calculated for the direct and indirect effects; an effect was considered statistically significant when its 95% CI did not include zero. All other tests were two-tailed, with P < 0.05 considered statistically significant.

## 3. Results

### 3.1. Descriptive statistics and population characteristics

Of the 504 residents included, 46.63% were male, and the most common age group was 20–25 years (56.75%). The sample was drawn from four hospitals and spanned multiple training specialties, including internal medicine, surgery, pediatrics, obstetrics and gynecology, general practice, otorhinolaryngology, and medical technology. Residents were classified into three categories by training pathway—clinical professional master’s, commissioned, and socialized—of which the socialized group was the largest (41.87%). By training year, 47.02% were in year one, 24.21% in year two, and 28.77% in year three. In addition, 25.99% had graduated from Project 211 universities, and 80.95% were unmarried. Full demographic characteristics are summarized in Table 1.

**Table 1:** Socio-demographic characteristics (n = 504).

Basic characteristics		N	%
Gender	Male	235	46.63
	Female	269	53.37
Age	20-25 years old	286	56.75
	26-30 years old	171	33.93
	>30 years old	47	9.32
Specialization in training	Internal Medicine	96	19.05
	Surgery	112	22.22
	Pediatrics	22	4.37
	Obstetrics and Gynecology	22	4.37
	General Practice	91	18.06
	Otorhinolaryngology	44	8.73
	Medical Technology	40	7.94
	Other	77	15.28
Types of resident physicians	Clinical professional masters	162	32.14
	Commissioned resident physician	131	25.99
	Socialized residents	211	41.87
The training hospital	The top hospital in the province	137	27.18
	The second-best hospital in the province	143	28.37
	The third-best hospital in the province	185	36.71
	Municipal hospital	39	7.74
Years of standardized residency training	First year	237	47.02
	Second year	122	24.21
	Third year	145	28.77
Highest degree	Undergraduate	341	67.66
	Postgraduate	163	32.34
Marital status	Single	408	80.95
	Married	96	19.05
Project 211 universities of graduation	Yes	131	25.99
	No	373	74.01
Medical licensing exam	Failed	196	38.89
	Passed	308	61.11

### 3.2. Perceived stress, burnout, ERI, and depressive symptom scores

The overall mean perceived stress score was 17.61 ± 6.36. Married residents scored higher than single residents (19.78 ± 7.44 vs. 17.10 ± 5.97), and residents older than 30 years had the highest scores (20.30 ± 8.72). The mean burnout score was 43.26 ± 13.04; graduates of Project 211 universities scored higher than non-Project 211 graduates (45.96 ± 12.88 vs. 42.31 ± 12.98), residents in the clinical professional master’s track scored higher than those in the other categories (47.20 ± 13.74), and trainees at the province’s leading hospital recorded the highest scores (45.18 ± 11.22), with scores decreasing at lower-tier hospitals. The mean ERI was 0.94 ± 0.65; scores were higher among postgraduates than undergraduates (1.00 ± 0.61 vs. 0.91 ± 0.67), among married than single residents (1.12 ± 0.81 vs. 0.90 ± 0.61), and among Project 211 than non-Project 211 graduates (1.02 ± 0.60 vs. 0.91 ± 0.67), and were highest among trainees at the province’s leading hospital (1.06 ± 0.54). The mean depressive symptom score was 7.83 ± 6.47; scores were higher among men than women (8.97 ± 7.20 vs. 6.83 ± 5.57), postgraduates than undergraduates (8.36 ± 5.85 vs. 7.58 ± 6.73), married than single residents (11.13 ± 8.47 vs. 7.05 ± 5.64), and Project 211 than non-Project 211 graduates (9.32 ± 6.71 vs. 7.31 ± 6.31). The highest depressive symptom scores were observed among residents older than 30 years (11.77 ± 8.10), trainees at the province’s leading hospital (9.27 ± 6.31), and third-year residents (9.68 ± 7.39). Group comparisons are shown in Table 2, Table 3, Table 4.

**Table 2:** Descriptive statistics of four continuous measurement variables.

	Mean	SD	Perceived Stress	Burnout	ERI	Depression
Perceived Stress	17.61	6.36	1			
Burnout	43.26	13.04	0.338**	1		
ERI	0.94	0.65	0.410**	0.568**	1	
Depression	7.83	6.47	0.363**	0.631**	0.608**	1

**P < 0.01

**Table 3:** Comparisons between perceived stress, burnout, ERI, and depression scores among different groups by Mann-whitney test.

	Perceived Stress		Burnout		ERI		Depression	
	mean ± SD	*P*	mean ± SD	*P*	mean ± SD	*P*	mean ± SD	*P*
**Gender**		0.175		0.428		0.232		0.002
Male	18.09 ± 6.81		43.83 ± 13.23		1.01 ± 0.77		8.97 ± 7.20	
Female	17.19 ± 5.91		42.77 ± 12.87		0.88 ± 0.52		6.83 ± 5.57	
**Highest degree**		0.214		0.063		0.009		0.018
Undergraduate	17.32 ± 6.57		42.56 ± 12.92		0.91 ± 0.67		7.58 ± 6.73	
Postgraduate	18.23 ± 5.86		44.73 ± 13.20		1.00 ± 0.61		8.36 ± 5.85	
**Marital status**		0.024		0.414		0.033		<0.001
Single	17.10 ± 5.97		42.91 ± 12.77		0.90 ± 0.61		7.05 ± 5.64	
Married	19.78 ± 7.44		44.73 ± 14.09		1.12 ± 0.81		11.13 ± 8.47	
**Project 211 universities**		0.076		0.003		0.024		<0.001
No	17.16 ± 6.14		42.31 ± 12.98		0.91 ± 0.67		7.31 ± 6.31	
Yes	18.90 ± 6.81		45.96 ± 12.88		1.02 ± 0.60		9.32 ± 6.71	
**Medical licensing exam**		0.102		0.076		0.773		0.452
Failed	18.42 ± 6.52		44.35 ± 12.55		0.93 ± 0.58		8.30 ± 6.86	
Passed	17.10 ± 6.21		42.56 ± 13.31		0.94 ± 0.70		7.53 ± 6.20	

**Table 4:** Comparisons between perceived stress, burnout, ERI, and depression scores among different groups by Kruskal Wallis test.

	Perceived Stress		Burnout		ERI		Depression	
	mean ± SD	*P*	mean ± SD	*P*	mean ± SD	*P*	mean ± SD	*P*
**Age**		0.022		0.160		0.053		0.001
20-25	17.38 ± 5.86		44.21 ± 12.69		0.90 ± 0.64		7.09 ± 5.94	
26-30	17.26 ± 6.26		41.51 ± 13.91		0.96 ± 0.67		7.98 ± 6.45	
>30	20.30 ± 8.72		43.83 ± 11.35		1.11 ± 0.66		11.77 ± 8.10	
**Types of resident physicians**		0.303		<0.001		0.106		0.232
Socialized residents	17.44 ± 7.07		41.78 ± 11.02		0.93 ± 0.65		8.06 ± 6.77	
Commissioned resident	17.33 ± 6.23		40.70 ± 14.28		0.88 ± 0.62		7.10 ± 6.41	
Clinical masters	18.07 ± 5.36		47.20 ± 13.74		1.00 ± 0.68		8.07 ± 6.08	
**The training hospital**		0.743		0.005	< 0.001		< 0.001	
Province’s leading hospital	18.30 ± 6.58		45.18 ± 11.22		1.06 ± 0.54		9.27 ± 6.31	
The second-best	17.71 ± 7.06		43.61 ± 13.67		0.92 ± 0.71		8.05 ± 7.06	
The third-best	17.05 ± 6.07		42.75 ± 13.79		0.93 ± 0.72		7.09 ± 6.13	
Municipal	17.51 ± 3.45		37.69 ± 11.51		0.65 ± 0.28		5.49 ± 5.17	
**Years of standardized residency training**		0.085		0.137		0.118		0.001
First year	17.97 ± 6.27		43.19 ± 12.96		0.94 ± 0.67		7.41 ± 6.40	
Second year	16.30 ± 5.77		41.32 ± 13.08		0.83 ± 0.46		6.44 ± 4.74	
Third year	18.12 ± 6.85		45.00 ± 12.97		1.03 ± 0.74		9.68 ± 7.39	

Overall, 139 participants (27.58%) scored ≥10 on the PHQ-9, indicating clinically significant depressive symptoms, and 194 (38.49%) scored ≥20 on the PSS-10, indicating high stress. Burnout classification identified no burnout in 85 participants (16.87%), mild burnout in 230 (45.63%), moderate burnout in 172 (34.13%), and high burnout in 17 (3.37%). In addition, 152 participants (30.16%) had an ERI > 1, indicating a high-effort–low-reward (imbalance) state.

### 3.3. Factors associated with depressive symptoms

Correlation analysis revealed significant positive associations (Table 2): burnout with perceived stress (ρ = 0.338); ERI with both perceived stress (ρ = 0.410) and burnout (ρ = 0.568); and depressive symptoms with perceived stress (ρ = 0.363), burnout (ρ = 0.631), and ERI (ρ = 0.608).

In multiple linear regression with depressive symptoms as the dependent variable, collinearity diagnostics showed tolerance values of 0.60–0.96 and variance inflation factors of 1.00–2.00, indicating no multicollinearity. After adjustment for demographic covariates (age and sex), four sequential models were constructed by incrementally adding perceived stress, burnout, and ERI (Table 5). In the adjusted models, perceived stress, burnout, and ERI were each significantly associated with depressive symptoms (all P < 0.001).

**Table 5:** Multiple linear regression of depression in resident physicians.

Independent variable	Model 1			Model 2			Model 3			Model 4		
	*β*	t	*P*	*β*	t	*P*	*β*	t	*P*	*β*	t	*P*
Gender	−0.165	−3.902	<0.001	−0.139	−3.639	<0.001	−0.119	−3.727	<0.001	−0.104	−3.440	0.001
Age	0.088	1.663	0.097	0.064	1.333	0.183	0.121	3.023	0.003	0.102	2.661	0.008
Types of resident physicians	0.021	0.463	0.643	0.002	0.055	0.956	−0.06	−1.753	0.080	−0.061	−1.859	0.064
The training hospital	−0.144	−3.270	0.001	−0.124	−3.126	0.002	−0.07	−2.100	0.036	−0.056	−1.770	0.077
Years of standardized residency training	0.160	3.195	0.001	0.151	3.346	0.001	0.100	2.662	0.008	0.112	3.116	0.002
Highest degree	0.020	0.453	0.651	0.001	0.028	0.978	−0.030	−0.903	0.367	−0.028	−0.875	0.382
Marital status	0.149	3.183	0.002	0.099	2.331	0.020	0.087	2.451	0.015	0.076	2.259	0.024
Project 211 universities of graduation	0.052	1.166	0.244	0.027	0.669	0.504	0.005	0.145	0.885	0.015	0.482	0.630
Medical licensing exam	−0.179	−3.529	<0.001	−0.127	−2.747	0.006	−0.105	−2.738	0.006	−0.121	−3.301	0.001
PSS—10				0.412	10.64	<0.001	0.255	7.522	<0.001	0.179	5.308	<0.001
MBI—GS							0.507	14.838	<0.001	0.381	10.366	<0.001
ERI										0.278	7.347	<0.001
F	9.269			21.558			48.326			53.566		
*P*	<0.001			<0.001			<0.001			<0.001		
Adjusted R^2^	0.129			0.290			0.509			0.556		
R^2^-changes	0.144			0.304			0.519			0.567		

### 3.4. Path model of the direct and mediating effects

**Fig 2:**
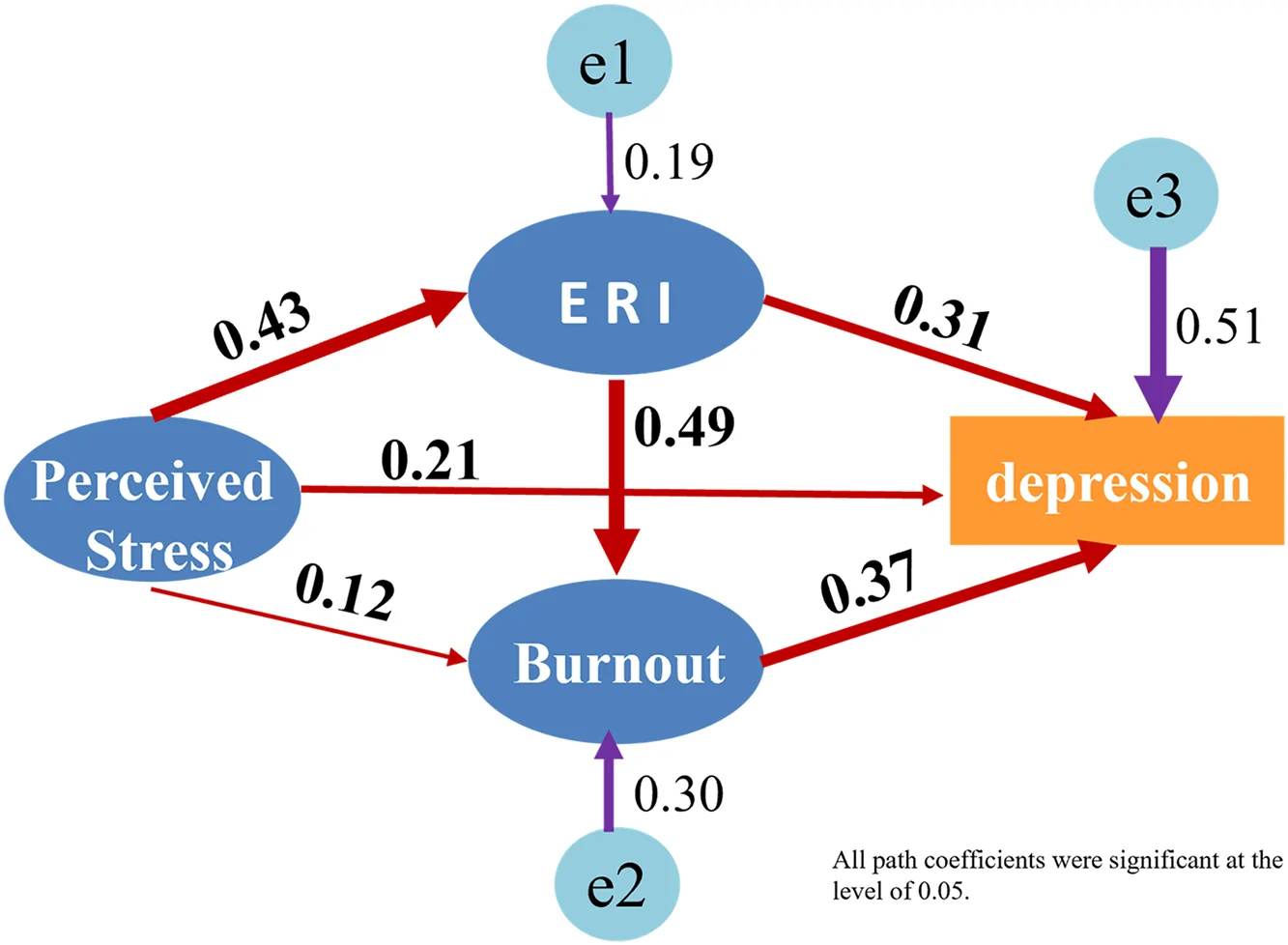
Standardized path coefficients for the just-identified path model of the relationships among perceived stress, ERI, burnout, and depression. All paths were statistically significant (P < 0.001).

A path model incorporating perceived stress, burnout, ERI, and depressive symptoms (Fig 2) was constructed to examine their interrelationships. Because the model included all directed paths among the four variables, it was just-identified (saturated; degrees of freedom = 0); global fit indices are therefore not informative for this type of model, and inference was based on the magnitude and statistical significance of the direct and indirect (mediation) effects, evaluated using bias-corrected bootstrapping. The standardized path coefficients were: perceived stress → ERI = 0.43; ERI → burnout = 0.49; perceived stress → burnout = 0.12; perceived stress → depressive symptoms = 0.21; ERI → depressive symptoms = 0.31; and burnout → depressive symptoms = 0.37. All paths were statistically significant (P < 0.001). Bootstrap analysis showed that the 95% CIs for the indirect effects excluded zero, indicating significant mediation: both burnout and ERI mediated the association between perceived stress and depressive symptoms. The 95% CIs for the direct effects also excluded zero, indicating significant direct associations alongside the mediated ones. Thus, burnout and ERI functioned as partial mediators, forming a serial mediation pattern within this model (Table 6).

**Table 6:** Bootstrap analysis of the significance test of the mediation effect.

Effect type	Effect size	SE	Bias-corrected 95%CI		*P*
			Lower	Upper	
Total effects	0.468	0.042	0.395	0.546	0.009
Direct effects	0.214	0.044	0.146	0.293	0.011
Total indirect effect	0.254	0.028	0.205	0.301	0.012
Ind1: perceived stress → ERI →depression	0.133	0.027	0.095	0.185	0.01
Ind2: perceived stress →burnout →depression	0.044	0.016	0.022	0.077	0.004
Ind3: perceived stress →ERI →burnout → depression	0.077	0.013	0.06	0.106	0.003

## 4. Discussion

This study characterizes the psychological burden experienced by physicians within the SRT system in a Chinese province. We found a high prevalence of perceived stress (38.49%), probable depression (27.58%), and burnout (83.13% with any degree of burnout, and 37.5% with moderate-to-severe burnout) in this cohort. In addition, our path model was consistent with ERI and burnout acting as statistical mediators. Situated within the distinctive stressors of China’s SRT system, these findings underscore the need for hospital administrators and medical educators to attend to the well-being of this key group and, in turn, to help safeguard the quality of patient care.

Residents reported high levels of perceived stress, with a mean score (17.61 ± 6.36) exceeding that reported in many general-population samples—a finding consistent with previous survey data on resident physicians [2,39]. Residents’ perceived stress is associated with the convergence of multiple institutional stressors. Chief among these is high workload intensity: extended working hours, heavy patient loads, demanding rotation schedules, and frequent night shifts persistently test physiological and psychological limits. For socialized residents (the largest subgroup), the absence of a formal employment contract during training and of guaranteed post-graduation employment can create profound anxiety about the future. Married residents (whose perceived stress scores were significantly higher than those of single residents) and residents older than 30 years (who had the highest scores) additionally face pressures from financial and social expectations, such as establishing families and supporting parents. Compounding these issues, the SRT system generally provides inadequate support for work–life balance.

The observed burnout prevalence of 83.13% is strikingly high, exceeding rates reported in a national survey of US psychiatry residents [40] while broadly aligning with findings from a study of residents in Guangdong, China [41]. Graduates of elite (Project 211) universities—25.99% of the sample—reported higher burnout, possibly reflecting greater expectations and perceived pressure to excel. Burnout scores tended to increase with hospital tier, peaking at provincial-leading hospitals; this suggests that burnout intensity is related to the workload and clinical environment of tertiary centers, the principal settings for SRT. The elevated burnout among residents in the clinical professional master’s track likely reflects the dual burden of intensive clinical training and demanding research and thesis requirements.

The mean ERI (0.94 ± 0.65) approached the threshold of 1.0, and 30.16% of residents already exhibited a high-effort–low-reward imbalance (ERI > 1). Although reported ERI prevalence among physicians varies widely (3.50%–96.9%, with a pooled prevalence of 40.2% for ERI > 1) [20], estimates specific to resident physicians typically cluster between 20% and 40% [13,42]. Residents expend considerable effort through long working hours, intense academic pressure, demanding clinical responsibilities, and the navigation of complex doctor–patient relationships, yet the rewards they perceive are often inadequate: financial compensation is generally low, and advanced qualifications do not yield commensurate remuneration (as reflected in the higher ERI among postgraduates and Project 211 graduates). The significantly elevated ERI among married residents (also highest in the > 30-year age group) likely reflects unmet financial and familial pressures compounded by insufficient systemic support.

The 27.58% prevalence of probable depression aligns with meta-analytic estimates (22.2%–28.8%) [17,43,44]. Significantly higher rates were observed in several subgroups: men, married residents, residents older than 30 years, third-year trainees, Project 211 graduates, and residents training at top-tier hospitals. Although baseline depression rates among incoming residents resemble those of age-matched peers in the general population [45], depressive symptoms rise markedly after residency begins. The peak in the third year is likely attributable to accumulated stress and to anxiety about impending career transitions—specifically, navigating a competitive job market (for socialized residents) or facing mandatory post-training assignments (for commissioned residents).

Our findings support both hypotheses. First, significant positive correlations were observed among all four core constructs—perceived stress, burnout, ERI, and depressive symptoms. The correlation coefficients (ρ = 0.338–0.631) indicate moderate-to-strong associations, consistent with prior research conducted internationally and in China [30–33]. Second, the path analysis indicated a serial mediation pattern: higher perceived stress was associated with higher ERI (β = 0.43), higher ERI with greater burnout (β = 0.49), and greater burnout with more severe depressive symptoms (β = 0.37). Perceived stress was also directly associated with depressive symptoms (β = 0.21), although the indirect (mediated) effects accounted for a substantial proportion of the total effect.

We situate this sequence within the transactional model of stress [46]. In this framework, perceived stress reflects a global primary appraisal that demands exceed available resources, whereas ERI represents a domain-specific secondary appraisal of the fairness of the effort–reward exchange at work. A heightened global stress state may lower the threshold for appraising specific work conditions as inequitable, providing a theoretical basis for modeling perceived stress as antecedent to ERI. We acknowledge, however, that within Siegrist’s model [14] ERI is often conceptualized as an occupational stressor that may itself precede perceived stress; the present cross-sectional design cannot adjudicate between these orderings, and the model should therefore be interpreted as one theoretically plausible pathway rather than a confirmed causal sequence.

Within this framework, the four constructs can be understood as successive appraisal-related stages. Perceived stress represents the initial appraisal, arising when demands exceed coping capacity—a common occurrence in SRT for the reasons outlined above. ERI reflects a subsequent, domain-specific appraisal, in which the effort expended (time, energy, and emotional labor) is perceived to outweigh the rewards received (salary, security, respect, and career prospects) [47,48]; in our data, higher perceived stress was strongly associated with higher ERI (β = 0.43). Burnout may represent an intermediate state through which persistent stress and a perceived unfair exchange are linked to an erosion of psychological resources [49]—depleted emotional energy (exhaustion), detachment and cynicism (depersonalization), and reduced feelings of competence (reduced personal accomplishment) [50]. In our model, burnout functioned as a statistical mediator, being associated with both perceived stress and ERI and, in turn, with depressive symptoms [40]. Depressive symptoms represent the downstream outcome of this pattern—anhedonia, hopelessness, fatigue, difficulty concentrating, and, in severe cases, suicidal ideation—with burnout showing the strongest (most proximal) association with depression among the variables examined.

Although SRT offers important benefits as a developmental pathway for medical trainees, the emergence of psychological problems threatens both personal health and patient safety. Enhancing residents’ engagement and performance requires addressing their core challenges; we therefore propose the following multilevel interventions. To address ERI, systemic measures are needed to reduce the effort–reward disparity: substantially increasing stipends and salaries, establishing clear pathways to stable post-training employment (especially for government-funded residents), improving working conditions (enforcing reasonable duty hours and ensuring adequate staffing), enhancing non-financial rewards (mentorship, recognition, respect, and meaningful feedback), and granting greater autonomy as competence develops.

For early intervention against burnout: because stress and ERI are associated with burnout, which is in turn associated with depression, mitigating burnout is critical [32]. This requires robust institutional support, including guaranteed rest and recovery time, accessible psychological support, and proactive attention to resident distress; the high burnout rates observed in top-tier hospitals point to a particular need for workload redistribution or enhanced support in these settings.

With respect to comprehensive stress management, because perceived stress is directly associated with depression and is also related to burnout and ERI, the early identification of residents’ distress is an urgent priority for researchers and healthcare administrators [51].Effective strategies include evidence-based individual interventions—such as cognitive behavioral therapy, mindfulness training, and time-management training—alongside organizational measures, enacted by administrators, to manage workload and scheduling and thereby reduce sources of stress.

In summary, our data underscore the need for multilevel, systemic interventions. At the policy level, formal SRT contracts should ensure livable wages, clearly defined rights and obligations, guaranteed employment pathways, and enforcement of reasonable duty hours with adequate staffing; increased national investment is also needed to optimize program structure, reduce burden, and enhance support. At the hospital level, a supportive culture is essential, with leaders modeling healthy behaviors and providing accessible mental health services, and with work environments and resource allocation optimized and targeted support offered to vulnerable groups. At the individual level, residents should be supported in strengthening their stress-management skills, fostering help-seeking behavior, and engaging with mentorship. This multipronged approach is essential for sustainable career development among residents.

### Limitations

This study has several limitations. First, participants were recruited through WeChat groups and voluntary online participation, which may introduce self-selection bias; residents with higher stress or depressive symptoms—or, conversely, those with more time and lower distress—may have been differentially likely to respond, potentially biasing prevalence estimates and limiting representativeness. The relatively low participation rate (about 32%) further increases this possibility, so the findings should be interpreted with caution and confirmed in probability-based samples. Second, all data were self-reported and thus susceptible to recall bias, social desirability bias (which may lead to underreporting of distress), and subjective interpretation; participants may also show a tendency toward acquiescence, although the anonymous format should have reduced the risk of respondents answering as they believed the researchers wished. Third, responses were restricted to a Likert-scale format, and qualitative methods might have provided deeper insight into participants’ perspectives and attitudes. Fourth, because the data are cross-sectional, the mediation pathways identified by the path model describe statistical associations consistent with the hypothesized model; they cannot establish temporal precedence or causality, and alternative orderings of the variables are equally compatible with the data, so longitudinal or experimental designs are needed to test directionality. Fifth, data collection was geographically restricted to southern China; although hospitals of varying tiers were included, the findings may not be fully generalizable to other regions. Finally, the provision of a small incentive and the low time commitment required may have introduced some participation bias.

## 5. Conclusion

This study characterizes the associations linking stress and depression among Chinese resident physicians and identifies effort–reward imbalance and burnout as key mediators. Supporting residents will require multilevel intervention: policy-level reforms to ensure fair contracts, livable wages, and enforced work-hour limits; hospital-level efforts to cultivate supportive cultures and provide accessible mental health resources; and individual-level support to help residents develop stress-management skills. This integrated approach is essential to address the factors linking stress and depression and to safeguard both physician well-being and the quality of patient care.

## Supporting information

S1 Checklist STROBE checklist. (DOCX)

S1 File Raw data. (XLSX)
