# Supplementary material for: How do young chinese physicians cope with stress under standardized training? Investigating perceived stress, depression, and their interrelationships
Source: PLoS One. 2026 Aug 24;21(8):e0356791. doi: 10.1371/journal.pone.0356791 (PMC13502695; doi:10.1371/journal.pone.0356791)
Supplement: Supplementary file 1 [file pone.0356791.s001.docx]

**STROBE Statement—Checklist of items that should be included in reports of *cross-sectional studies***

Manuscript: How Do Young Chinese Physicians Cope with Stress Under Standardized Training? Investigating Perceived Stress, Depression, and Their Interrelationships (PONE-D-26-01680)

|  | Item No. | Recommendation | Reported in manuscript (section / location) |
| --- | --- | --- | --- |
| **Title and abstract** | 1 | (a) Indicate the study’s design with a commonly used term in the title or the abstract | Abstract, *Methods* (“multicenter cross-sectional study”); Methods 2.1 |
|  |  | (b) Provide in the abstract an informative and balanced summary of what was done and what was found | Abstract (Background, Methods, Results, Conclusions) |
| **Introduction** |  |  |  |
| Background/rationale | 2 | Explain the scientific background and rationale for the investigation being reported | Introduction, paragraphs 1–4 |
| Objectives | 3 | State specific objectives, including any prespecified hypotheses | Introduction, final paragraph (“this study aims to…”; hypotheses 1 and 2) |
| **Methods** |  |  |  |
| Study design | 4 | Present key elements of study design early in the paper | Methods 2.1 (Study design) |
| Setting | 5 | Describe the setting, locations, and relevant dates, including periods of recruitment, exposure, follow-up, and data collection | Methods 2.2 (Setting: Wenjuanxing/Sojump platform, April 1–20 2025, four hospitals in southern China); 2.3 |
| Participants | 6 | (a) Give the eligibility criteria, and the sources and methods of selection of participants | Methods 2.3 (Participants and enrollment); Figure 1 |
| Variables | 7 | Clearly define all outcomes, exposures, predictors, potential confounders, and effect modifiers. Give diagnostic criteria, if applicable | Methods 2.4.1–2.4.5 (depressive symptoms, perceived stress, burnout, ERI; scoring thresholds); demographic covariates in 2.4.1 |
| Data sources/measurement | 8 | For each variable of interest, give sources of data and details of methods of assessment (measurement). Describe comparability of assessment methods if there is more than one group | Methods 2.4.1–2.4.5 (PHQ-9, PSS-10, MBI-GS, ERI: items, scoring, cut-offs, reliability/validity) |
| Bias | 9 | Describe any efforts to address potential sources of bias | Methods 2.2 (exclusion of careless/“speeded” responses <200 s); Discussion–Limitations (self-selection, self-report, social desirability) |
| Study size | 10 | Explain how the study size was arrived at | Methods 2.5 (Sample size: participant-to-parameter ratio, bootstrap power analysis); 2.3 (proportional allocation across sites) |
| Quantitative variables | 11 | Explain how quantitative variables were handled in the analyses. If applicable, describe which groupings were chosen and why | Methods 2.4 (categorization thresholds: PHQ-9 ≥10; PSS-10 low/moderate/high; burnout classification; ERI >1); 2.6 |
| Statistical methods | 12 | (a) Describe all statistical methods, including those used to control for confounding | Methods 2.6 (Shapiro–Wilk; Mann–Whitney U; Kruskal–Wallis H; Spearman correlations; multiple linear regression adjusted for covariates; path model with bootstrap) |
|  |  | (b) Describe any methods used to examine subgroups and interactions | Methods 2.6; Results 3.2 (group comparisons by demographic variables); Tables 3–4 |
|  |  | (c) Explain how missing data were addressed | Methods 2.2 (only complete, valid questionnaires retained; incomplete/invalid submissions excluded) |
|  |  | (d) If applicable, describe analytical methods taking account of sampling strategy | Methods 2.3 (proportional allocation by number of residents per site) |
|  |  | (e) Describe any sensitivity analyses | Results 3.3 (sequential/hierarchical regression models, Table 5); collinearity diagnostics (tolerance, VIF) |
| **Results** |  |  |  |
| Participants | 13 | (a) Report numbers of individuals at each stage of study—eg numbers potentially eligible, examined for eligibility, confirmed eligible, included in the study, completing follow-up, and analysed | Methods 2.2 (≈1,756 invited; 561 submitted; 57 excluded; 504 analysed); Figure 1; Results 3.1 |
|  |  | (b) Give reasons for non-participation at each stage | Methods 2.2 (57 excluded for completion time <200 s; participation rate ≈32%) |
|  |  | (c) Consider use of a flow diagram | Figure 1 (Questionnaire collection flowchart) |
| Descriptive data | 14 | (a) Give characteristics of study participants (eg demographic, clinical, social) and information on exposures and potential confounders | Results 3.1; Table 1 |
|  |  | (b) Indicate number of participants with missing data for each variable of interest | Not applicable—only complete questionnaires were analysed (Methods 2.2) |
| Outcome data | 15 | Report numbers of outcome events or summary measures | Results 3.2 (prevalence: probable depression 27.58%, high stress 38.49%, burnout 83.13%, ERI imbalance 30.16%); Tables 2–4 |
| Main results | 16 | (a) Give unadjusted estimates and, if applicable, confounder-adjusted estimates and their precision (eg, 95% confidence interval). Make clear which confounders were adjusted for and why they were included | Results 3.3 (multiple linear regression adjusted for age and sex, Table 5); 3.4 (standardized path coefficients and bootstrap 95% CIs, Table 6) |
|  |  | (b) Report category boundaries when continuous variables were categorized | Methods 2.4 (cut-offs); Results 3.2 |
|  |  | (c) If relevant, consider translating estimates of relative risk into absolute risk for a meaningful time period | Not applicable (cross-sectional; associations reported, no relative-risk estimates) |
| Other analyses | 17 | Report other analyses done—eg analyses of subgroups and interactions, and sensitivity analyses | Results 3.4 (path/mediation analysis, Table 6); subgroup comparisons (Tables 3–4) |
| **Discussion** |  |  |  |
| Key results | 18 | Summarise key results with reference to study objectives | Discussion, paragraph 1 |
| Limitations | 19 | Discuss limitations of the study, taking into account sources of potential bias or imprecision. Discuss both direction and magnitude of any potential bias | Discussion–Limitations (self-selection, self-report/social desirability, cross-sectional design, geographic restriction, unmeasured confounders) |
| Interpretation | 20 | Give a cautious overall interpretation of results considering objectives, limitations, multiplicity of analyses, results from similar studies, and other relevant evidence | Discussion (interpretation within the transactional model of stress; comparison with prior studies; associations not causal) |
| Generalisability | 21 | Discuss the generalisability (external validity) of the study results | Discussion–Limitations (recruitment via WeChat; southern-China sites; findings to be confirmed in probability-based samples) |
| **Other information** |  |  |  |
| Funding | 22 | Give the source of funding and the role of the funders for the present study and, if applicable, for the original study on which the present article is based | Funding Statement (Hunan Medical Scientific Research Project W20243020; Natural Science Foundation of Hunan Province 2025JJ50449) |
